# Cholecalciferol level and its impact on COVID-19 patients

**DOI:** 10.1186/s43162-022-00116-w

**Published:** 2022-02-21

**Authors:** Mohammed Abdel Monem Saeed, Alaa Hussein Mohamed, Ahmed Hassan Owaynat

**Affiliations:** 1 Critical Care Medicine Department, Faculty of Medicine Helwan University, Helwan, Egypt; 2 Clinical Pharmacy Department, Helwan University Hospitals, Helwan, Egypt; 3 Nursing Department Helwan University Hospitals, Helwan, Egypt

**Keywords:** Vitamin D_3_ (cholecalciferol), COVID-19, Cytokine storm markers, Chest computed tomography, Mortality

## Abstract

**Background:**

Cholecalciferol is an important nutrient and essential to build body, maintain strong bones, and improves immunity.

The main source for vitamin D is the body’s skin which absorbs the sun’s ultraviolet rays and convert them into vitamin D; at the same time, deficiency can occur or people may not get enough supplementation; this occurs mainly in old age, not taking healthy food, or have darker skin, and this deficient cases can raise the risk of severe COVID-19 if infected.

Vitamin D boosts immunity and decreases inflammation. Poorer outcome of corona virus—disease (COVID-19) has been suggested to be due to vitamin D deficiency.

We suggested to find the effect of cholecalciferol levels 25-hydroxy vitamin D (25 OHD) on the severity and mortality in patients suffering from COVID-19.

**Methods:**

Our study is a prospective following of 414 patients admitted in Helwan University Hospitals in the period of June 2020 till October 2021 for severely symptomatic. COVID-19 patients with median of age 54.55 ± 14.27, with a definite range of APACHE II score ranging from 15 to 19 where we measured vitamin D_3_ level (cholecalciferol level), correlating the assay level to the inflammatory cytokine storm markers on admission, on the fifth day and after 10 days also the level of vitamin D_3_ was correlated to the length of stay mechanical ventilation days and mortality.

**Results:**

Lower level of vitamin D_3_ on admission was strongly evident in patients with severely symptomatic and in mortality of COVID-19 patients 58.25 ± 24.59 nmol/L when compared with patients who survived 103.97 ± 36.14 nmol/L with *P* value < 0.001.

Also, when correlating the initial level of vitamin D_3_ on admission with the level of the inflammatory cytokine storm markers on admission, on fifth day from admission and on the tenth day, it shows a strong inverse correlation between vitamin D_3_ level on admission and ferritin level on fifth day ρ–0.739 *p* value < 0.001 also on the tenth day ρ–0.885, *P* value < 0.001, in comparing also with D-dimer on fifth day ρ–0.858, *p* value < 0.001 also showing a strong inverse correlation with a highly significant *p* value this also evident on the D-dimer level on the tenth day ρ–0.889 with *p* value < 0.001, CRP at fifth and tenth day ρ–0.868, *P* value < 0.001, ρ–0.891, *P* value < 0.001 respectively also in correlating the LDH level on the fifth and tenth day with the initial level of vitamin D_3_ it shows a strong inverse correlation with a highly significant *p* value. ρ–0.887, *P* value < 0.001, ρ–0.878, *p* value < 0.001 respectively, in the fifth and tenth day. Neutrophil to lymphocyte ratio was strongly, inversely correlated to the vitamin D_3_ level (cholecalciferol) on admission with ρ–0.753, *p* < 0.001, ρ–0.882, *P* < 0.001 respectively. Also, chest computed tomography in the fifth and tenth day of admission showed a very strong inverse correlation with vitamin D level and a highly significant statistical difference ρ–0.655, *p* value < 0.001 respectively.

Length of stay and mechanical ventilation days were also strongly inversely correlated to the cholecalciferol level ρ–0.795, *p* < 0.001, ρ– 0.879, *P* < 0.001 ROC curve of vitamin D_3_ to predict mortality (RR 0.865, 95% CI 0.828–0.896, *P* < 0.001, with cut off-value for vit. D_3_ < 60 nmol/L, regardless of other factors like age, gender, and presence of other co-morbidities.

**Conclusion:**

Low level of cholecalciferol was strongly inversely correlated with cytokine storm markers and independent predictor of severity and mortality in COVID-19 patients.

## Background

Millions of individuals have been killed by SARS-Cov 2 leading to a great depression; also, it broadly varies from benign to fatal. Severe corona virus disease can be presented by interstitial pneumonia progressing to acute respiratory distress syndrome (ARDS) and respiratory failure ending in death or other co-morbidities. Pneumonia can progress to severe acute respiratory failure which will need invasive mechanical ventilation with prevalence up to 95% of respiratory distress this is evident in patients who died from the disease [1].

The imbalance of the host immune system is the main determinant of the SARS-Cov-2 outcome [2–4].

Positive effect against infection exerted by the immune response facilitates viral clearance this is exerted by the primary immune response while the secondary immune response may lead to exaggerated response leading to multiple organ failure, ARDS, and death [2].

Predictors of COVID-19 mortality mainly include elevated cytokine levels or other inflammatory markers [5], so mortality may be related to virally driven-hyper inflammation known as “Cytokine Storm” [3, 4].

Vitamin D (25-hydroxy vitamin D) lately identified as a potential effective drug in the prevention, and treatment of COVID-19 [2].

25 OHD has been epidemiologically linked to many health outcomes [3, 4]. An immunomodulatory role has been found for the vitamin D, so it became a target in COVID-19 management.

Thirty-seven percent of elderly adults in the USA take vit D supplements [6, 7]. Also, it provides a way to lessen burden of the SARS-Cov pandemic.

Some RCTS shown a benefit of 25 OHD supplementation [7]. A meta-analysis of RCTs of 25 OHD supplementation showed benefit in the benign upper respiratory tract and not the lower respiratory tract (OR = 0.96; 95% CI: 0.83, 1.10) and showed numerically worse all-cause mortality (OR = 1.39; 95% CI: 0.85, 2.27).

In the pandemic of COVID-19, due to the complexity of the pathophysiology of the disease, and due to the deficiency of supported data this can lead to biased information. One of the debatable issues is the vitamin D role in modulating the severity of COVID-19, as this vitamin has an immunomodulatory functions extending from the innate to the adaptive arms of the immune system including the downregulation of pro-inflammatory cytokines [6].

A lot of clinical and preclinical observations suggested that low vitamin D levels will be in favor of viral infections especially the respiratory tract infections, and the autoimmune disorders [6, 7].

Also, there is a lot of studies shown that vitamin D supplementation protect against respiratory infection [8, 9] and there is a strong relation between vitamin D deficiency [levels below 20 ng/ml] and increased risk of progression ad death from viral infections like HIV, because of immune activation, severe inflammation, and monocyte phenotypic activation [9].

An independent relationship become evident from comorbid conditions, this occurs in HIV determining the severity and mortality [10].

The Scientific Advisory Committee on Nutrition (SACN) from UK suggested that there is a benefit from daily low-dose vitamin D. Supplementation (400 to 1000 Iu/day) in protection from acute respiratory tract infections [11].

So, to strengthen the immune system and to help in fighting against COVID-19, vitamin D plays a great role in that [12].

This concept is supported by recent epidemiological data, suggesting a strong relationship between COVID-19 severity and vitamin D deficiency [13–15].

Also there are some reports in patients with or without COVID-19 supporting the previous data [16–22].

It needs to be detected on a prospective way, and it should be taken into consideration whether vitamin D deficiency or low 25OHD levels, at time of diagnosis, is associated with the severity of COVID-19 and not other confounding factors like age, gender, and major comorbidities. So, a demonstration is required for further support of issuing clinical trials to help in assessing the efficacy of vitamin D level, in patients infected with SARS-Cov.2. So, we initiated our study to investigate prospectively in a cohort of COVID-19 patients admitted in our ICU, the relationship between 25OHD levels at ICU admission and severity of COVID-19 and related mortality during the ICU course.

## Methods

Four hundred fourteen COVID-19 patients, diagnosed as a positive result from naso-pharyngeal swab associated with reverse transcription-polymerase chain reaction analysis (Gene X pert, Cepheid, Sunnyvale, USA), were admitted to the ICU with critical COVID-19 pneumonia with acute respiratory failure (spontaneous oxygen saturation ≤ 93%, and/or PaO_2_/FiO_2_ ratio < 300 mmHg) requiring invasive mechanical ventilation, with or without fever and other organ dysfunction in Helwan university hospitals in the period from June 2020 till October 2021. Vitamin D_3_ level was assayed in all admitted patients with COVID-19 pneumonia in the ICU, and vitamin D deficiency was defined in each patient with 25OHD ≤ 60 nmol/L.

All patients were subjected to full laboratory investigations including the cytokine inflammatory markers ferritin, D-Dimer, LDH, CRP, and neutrophil: lymphocyte ratio on admission, on fifth and tenth day of ICU admission, daily arterial blood gas, daily chest X-ray, and chest computed tomography on admission, fifth day, and tenth day from admission.

### Ethical approval

The study was approved by the ethical committee of faculty of Medicine Helwan University. It was conducted in compliance with local regulatory requirements, good clinical practice (GCP), and the Declaration of Helsinki.

### Statistics

The collected data was revised, coded, tabulated and introduced to a pc using statistical package for social science (SPSS 25). Data was presented and suitable analysis was done according to this type of data obtained for each parameter.

#### Descriptive statistics

1. Mean, standard deviation (± SD) and range for parameteric numerical data, while median and interquartile range (IQR) for non-parameteric numerical data.
2. Frequency and percentage of non-numerical data.

#### Analytical statistics

1. Student’s *t* test was used to assess the statistical significance of the difference between two study group means.
2. Mann-Whitney test (*U* test) was used to assess the statistical significance of the difference of a non-parametric variable between two study groups.
3. Correlation analysis (using Pearson’s and Spearman’s rho methods) to assess the strength of association between two quantitative variables. The correlation coefficient denoted symbolically “*r*” defines the strength (magnitude) and direction (positive or negative) of the linear relationship between two variables.
  *r* = 0–0.19 is regarded as very weak correlation.
  *r* = 0.2–0.39 as weak correlation.
  *r* = 0.40–0.59 as moderate correlation.
  *r* = 0.60–0.79 as strong correlation.
  *R* = 0.80–1 as very strong correlation.
4. The ROC curve (receiver operating characteristic) provides a useful way to evaluate the sensitivity and specificity for quantitative diagnostic measures that categorize cases into one of two groups *p* value:
  level of significance:
  *P* > 0.05: non-significant (NS).
  *P* < 0.05: significant (S).

## Results

Four hundred and fourteen COVID-19 pneumonia patients admitted to our ICU were enrolled in our clinical trial in the period from June 2020 to October 2021 (Table 1).

**Table 1:** Demographic status of the study (*N* = 414)

	N/Mean	%/SD	Median (DR)	Range
Age	54.55	14.27	55 (45–64)	(14–97)
Gender				
Male	285	68.8%		
Female	129	31.2%		

Table 2 shows the correlation between vit D_3_ level and laboratory investigations especially the cytokine storm markers on the fifth and tenth days, which shows a very strong inverse correlation with vitamin D_3_ level and a highly significant statistical difference. While the cytokine storm markers on admission ferritin, D-dimer, CRP, LDH, and neutrophil to lymphocytes ratio were all non-significant statistically with a very weak correlation. Also, chest computed tomography revealed a strong and very strong inverse correlation with vitamin D level on admission, fifth day and tenth day respectively with a highly significant statistical difference on days five and ten of admission.

**Table 2:** Correlation between vitamin D_3_, laboratory investigations and chest computed tomography

		**Ferritin admission**	**Ferritin + 5 days**	**Ferritin + 10 days**	**D-dimer admission**
**Vitamin D**_**3**_	**Spearman’s rho**	− 0.015	− 0.739	− 0.885	− 0.046
	***P*value**	0.758	< 0.001	< 0.001	0.347
	**Sig.**	NS	H.S.	H.S.	NS
**D-dimer + 5 days**	**D-dimer + 10 days**	**CRP admission**	**CRP + 5 days**	**CRP + 10 days**	**LDH admission**
− 0.858	− 0.889	0.95	− 0.868	− 0.891	0.022
< 0.001	< 0.001	0.005	< 0.001	< 0.001	0.661
H.S.	H.S	NS	H.S	H.S	NS
**LDH** **+ 5 days**	**LDH** **+ 10 days**	**N:L ratio admission**	**N: L ratio** **+ 5 days**	**N:L ratio** **+ 10 days**	
− 0.887	− 0.878	− 0.075	− 0.753	− 0.882	
< 0.001	< 0.001	0.26	< 0.001	< 0.001	
H.S.	H.S.	H.S.	H.S.	H.S.	
**CT-chest admission**		**CT-chest** **+ 5 days**		**CT-chest** **+ 10 days**	
− 0.655		− 0.876		− 0.916	
0.05		< 0.001		< 0.001	
S		H.S.		H.S.	

Table 3 shows a very strong inverse correlation of MV days and vitamin D_3_ level with a highly significant statistical value.

**Table 3:** Correlation between vitamin D_3_ level and mechanical ventilation days

	MV days
Vitamin D_3_ level	
Spearman’s rho	− 0.879
*P* value	< 0.001
Sign.	H.S.

Table 4 shows a very strong inverse correlation between vitamin D_3_ level and length of stay with a highly significant statistical value.

**Table 4:** Correlation between vitamin D_3_ level and length of stay

	Length of stay
Vitamin D_3_ level	
Spearman’s rho	− 0.795
*P* value	< 0.001
Sign.	H.S.

Table 5 shows the relation between vitamin D_3_ level and the destination (outcome) of COVID patients according to vitamin D_3_ level where in the expired patients, the level of vitamin D_3_ was low giving a highly significant statistical difference between the expired and the alive outcome.

**Table 5:** Relation between vitamin D_3_ level and destination

	Vitamin D_**3**_ level		Student’s ***t*** test		
	Mean + SD	Median (I Q R)	T	***p*** value	Sign.
Destination					
Expired	58.25 + 24.59	54 (44–65)	− 15.195	< 0.001	H.S.
Discharged alive	103.97 ± 36.14	107.5 (74–132)			

The ROC curve of vitamin D_3_ with sensitivity 94.09 and specificity 67.53, cut-off value of vitamin D_3_ to predict mortality is less than 60 nmol/L. Area under the curve was 0.865, 95% CI 0.828–0.896 (Table 6).

**Table 6:** Receiver operator characteristic curve of vitamin D_3_ to predict mortality

AUC	95% CI	Sign.	Cut-off value	Sensitivity	Specificity
0.865	0.828–0.896	< 0.001	< 60	94.09	67.53
+ LR	− LR	+ PV	− PV		
2.9	0.088	76.7	91		

## Discussion

SARS-Cov.2 pandemic with its higher morbidity and mortality rates, pushed us to study the actual mechanisms leading to the poor clinical outcomes in the trial to find effective treatments [1].

For that 25OHD was measured for assaying its levels in a series of SARS–Cov-2-infected patients with severely symptomatic COVID-19 cases who admitted to the ICU with respiratory failure.

For patients who deteriorated and did not improve, the vitamin in D_3_ level was low leading to long length of stay, prolonged period of mechanical ventilation days, and ending by death while patients with normal level or mild deficiency improved along the ICU course with improving cytokine storm markers, also clinically through the evaluation done on the fifth and tenth days of ICU stay, leading to less duration of ICU stay, mechanical ventilation days and at the end they survived. This over all data matches with similar studies done previously [16–22], and emphasis on that vitamin D deficiency is independently associated with the clinical outcome of COVID-19 patients away from age or other comorbidities commonly detected in admitted COVID-19 patients.

A possible correlation was suggested in many different studies between vitamin D_3_ levels and COVID-19 on indirect proofs like the risk of SARS–COV 2 infection and its spread [14] or the inverse correlation between vitamin D_3_ levels mean and the number of infected cases in Europe [15]. Many large observational studies, and also cohort studies found significantly lower levels of 25OHD in COVID-19 patients when compared with negative cases of COVID, suggesting a strong relation between low 25 OHD levels and COVID-19 positivity [23–26].

The results of our study are in line with all previous observational studies. Suggesting that low level of 25OHD levels may lead to infection with SARS-Cov-2 and leads to risk of severe respiratory failure and mortality during the ICU course in patients affected by SARS–Cov-2.

There is a strong inverse correlation between vitamin D level and the cytokine storm inflammatory markers like ferritin, D-dimer, CRP, LDH, and neutrophil to lymphocyte ratio especially in the fifth and tenth days of the ICU course of patients affected by COVID-19 [27–29].

Low vitamin D is associated with increased inflammatory reaction in patients taking bisphosphonate and became more evident when vitamin D level is below 60 nmol/L [30].

Experimental studies found that, vitamin D locally activated in lung tissues and leads to beneficial effect on interstitial pneumonia experimentally [31, 32].

Also many mechanisms can be involved in the protection of lung tissue like modulation of neutrophil activity by decreasing their activation and penetration into the lung tissue, so decreasing alveolar damage, it can also occur by suppressing all the pro-inflammatory cytokines, also activation of cathelicidin and defesins which can destruct the virus itself [7, 15].

In Experimental animals vitamin D maintain, the pulmonary vascular barrier from jury by inflammatory process by affecting the renin angiotensin system [33], which can lead to invasion of the alveolar cells by the COVID-19 virus with activation of inflammatory cytokines and occurrence of ARDS [2, 34].

Vitamin D also plays a major role in activation of surfactant synthesis, improving alveolar surface tension, protecting against inflammation, and decreasing oxidative stress [35].

So, normal vitamin D can improve clinical outcome of SARS-Cov-2 by maintaining pulmonary vascular barrier and aborting the release of the inflammatory cytokine storm markers.

From the epidemiological data it shows that death from SARS-Cov-2 infection was more in the countries which has more winter time because vitamin D deficiency is more prominent in winter season and because of lack of supplementation policies [36].

In the start of pandemic, in the first quarter of the year 2020, there is a larger spread in the northern hemisphere along the 30–50^o^N latitude zone while it was less by around 2–6% in the courtiers below the 37^o^N parallel [37] because of good sun exposure leading to adequate 25 OHD levels even if there is fall or in winter time [6], and so death rate was lower in those countries.

Also, flows of the pandemic in the northern countries showed an increase in mortality starting from the last three months of 2020 as it is winter time; US SARS-Cov-2 mortality was 4–5 times more among black citizen patients, whom generally have lower vitamin D concentration than other citizens [38, 39].

Vitamin D levels were higher in the surviving patients who experienced COVID-19 even if they experienced mechanical ventilation and ARDS in comparing with those who died as their vitamin D levels were very low; also cytokine storm markers starts to regress starting from fifth and tenth day post-ICU admission giving a very strong inverse correlation with the vitamin D level in the surviving patients leading to clinical improvement and weaning from mechanical ventilation.

Vitamin D levels found in many studies to be low in severely symptomatic COVID-19 patients which can be considered as an acute phase reactant and can decrease with the severity of the disease (https://www.aifa.gov.it/-/vitamina-d-consumi-e-spesa-ridotti-dall-introduzione-della-nota-96), [40–43], which also was confirmed in many observational studies where they found a strong relation between 25 OHD levels and the severity of SARS–Cov-2 [26, 44–46].

Also, recent data from UK Biobank, where 1082 SARS-Cov-2 patients were included showing a strong relationship between vitamin D level and severity of the case of COVID-19-affected patients [47–49].

All the previous studies confirmed the results of our study that the level of vitamin D is very strongly inverse correlated with the severity of the case of COVID-19 as regards the cytokine storm markers which was followed along the ICU course in the fifth and tenth days, and improved in the surviving patients leading to less mechanical ventilation days and less length of stay.

## Conclusion

Vitamin D levels plays a crucial role in increasing or decreasing the level of the cytokines storm inflammatory markers according to the actual level of vitamin D; if there is deficiency and the level is low, the cytokine storm inflammatory markers are high and patient experiences a severely symptomatic COVID-19 affection and becomes an independent predictor of mortality other than any co-morbidities and can be a prognostic marker of the severity of the COVID-19-diseased patients, so vitamin D supplementation during the course of COVID-19-affected patients can affect the progression of the severity of the disease [50–56].

## Data Availability

Available upon request.
